# Factors and interventions determining the functioning of health care teams in county-level hospitals in less affluent areas of China: a qualitative study

**DOI:** 10.3389/fpubh.2023.1082070

**Published:** 2023-09-28

**Authors:** Hujie Wang, Jeroen van Wijngaarden, Martina Buljac-Samardzic, Joris van de Klundert

**Affiliations:** ^1^Erasmus School of Health Policy and Management, Erasmus University Rotterdam, Rotterdam, Netherlands; ^2^School of Business, Universidad Adolfo Ibáñez, Santiago, Chile

**Keywords:** teamwork, team functioning, team interventions, leadership, multidisciplinary team, county-level hospitals, less affluent areas, China

## Abstract

**Background:**

Teamwork is essential for the quality and safety of care, and research on teamwork in health care has developed rapidly in many countries. However, evidence from less affluent, non-Western countries is scarce, while improving teamwork may be especially relevant to be able to increase the quality of care in these settings. This study aims to understand the main factors that influence, and interventions used to improve, the functioning of health care teams in the context of county-level hospitals in less affluent areas of China.

**Methods:**

We conducted semistructured interviews to explore the factors that influence team functioning and the interventions implemented to improve team functioning in these hospitals. 15 hospital presidents and 15 team leaders were selected as respondents.

**Results:**

From the interviews, we have identified five main factors that influence team functioning in these hospitals: “stuck in the middle”, local county setting, difficulty in attracting and retaining talent, strong focus on task design, and strong focus on leadership. The interventions for improving team functioning can mostly be categorized as the following: 1) measures to attract and retain talent (e.g., increase salary, train talent in national or provincial level hospitals, and provide fast-track promotions), 2) interventions focused on monodisciplinary teams (e.g., changing the team structure and leadership, and skill training), and 3) interventions to establish and improve multidisciplinary teams (e.g., simulation training and continuous team process improvements).

**Conclusion:**

With the introduction of multidisciplinary teams, interventions into team processes have started to receive more attention. The findings depict an overview of the main factors and interventions as specifically relevant for team functioning in county-level hospitals in less affluent areas of China and may help these hospitals benefit from additional process interventions to improve teamwork and the quality of care.

## Introduction

Health care is a highly demanding industry, which requires effective teamwork to provide high-quality care for patients. The landmark publication “To err is human” has pointed out the key role of teamwork in reducing medical errors (1). Since then, the evidence base supporting the impact of teamwork on the quality and safety of care has continued to grow. Manser’s (2) review confirms that teamwork plays a key role in preventing adverse events. Schmutz et al.’s (3) systematic review and meta-analysis more generally shows that teamwork is positively related to the clinical performance of health care teams.

Due to the importance of teamwork in the quality and safety of care, research on the functioning of health care teams has been blooming in recent decades. Lemieux-Charles & McGuire have proposed the Integrated (Health Care) Team Effectiveness Model (ITEM) to describe the relationships among team inputs (i.e., social and policy context, organizational context, and task design), processes, and outcomes (4). This model is the foundation and starting point of many teamwork studies in health care. Other researchers have focused on interventions to improve team functioning in health care. For example, Buljac-Samardzic et al. (5) have proposed four categories of such interventions in health care: training, tools, (re)design, and combinations of interventions from multiple categories. McCulloch et al. (6) have reviewed the effects of teamwork training on health care professionals’ performance and found enhanced teamwork after training, as well as improved staff attitudes, efficiency, and reductions in medical errors.

The existing evidence on teamwork in health care is, however, mostly from Western countries. Evidence from less affluent, non-Western contexts is especially lacking. This can be viewed as problematic in contexts such as less affluent Chinese areas, as the World Bank and the World Health Organization have advocated enhancing teamwork in Chinese hospitals as one of the strategies to improve the quality of care delivered by Chinese hospitals (7). This calls for research to increase the evidence base and close the knowledge base by exploring the functioning of health care teams and the interventions for improving team functioning in these hospitals, which may also be relevant for other less affluent, non-Western areas in the world.

A recent review of the evidence on teamwork in Chinese hospitals finds that most of the included studies were conducted in national and provincial level hospitals and that the evidence base for county-level hospitals and primary care institutions is scarce (8). The 17,294 county-level hospitals play a pivotal role in the Chinese health system (9). Positioned between primary care institutions on the one hand and national and provincial level hospitals on the other hand, they are required to provide an extensive variety of health services for the population of more than 498 million living in counties and county-level cities (10).

Governmental authorities and populations of counties and county-level cities in less affluent areas of China often face resource shortages that can negatively impact the health services delivery infrastructure, particularly for county-level hospitals. Thus, county-level hospitals in less affluent areas face unique context-specific challenges. The validity of existing evidence on teamwork in China’s national and provincial level hospitals in affluent areas may therefore be limited for this context. In this study, we aim to extend the understanding of team functioning and team interventions in these hospitals. More specifically, we propose the following two research questions:

(1) What are the main factors that influence the functioning of health care teams in county-level hospitals in less affluent areas of China?
(2) What interventions have been implemented by county-level hospitals in less affluent areas of China to improve the functioning of health care teams?

## Materials and methods

### Research method

The ITEM shows that social and policy context plays an indispensable role in team functioning, as is further emphasized by the Context-Interventions-Mechanisms-Outcome logic that explicitly captures the role of context in understanding the effects of interventions on outcomes (4, 11). Because of the scarce evidence on team functioning and team interventions in the context at hand, i.e., county-level hospitals in less affluent areas of China, and the limited validity of existing evidence in this unique context, it is necessary to construct our understanding of factors influencing team functioning and interventions to improve functioning specifically in this context rather than assuming that the known factors from research are valid. Therefore, our study is of explorative, phenomenological nature, following the constructivist paradigm and using semi-structured interviews for data collection (12–14). The reporting of this study follows the Standards for Reporting Qualitative Research (SRQR) guideline (15). The SRQR checklist is attached as Supplementary File S1.

This study was approved by the Research Ethics Review Committee of Erasmus School of Health Policy and Management, Erasmus University Rotterdam (Approval No. 21–035). Oral informed consent was obtained from all the participants before the data collection.

### Interview topics

The interviews have two parts. The first part addresses the factors that influence team functioning, and the second part considers the interventions implemented to improve team functioning. Each part includes both general, open-ended questions, and more structured questions based on a list of topics extracted from the literature. The interview guide is presented in Supplementary File S2.

The topic list for the first part is rooted in an input-process-outcome-based teamwork model as also adopted in the aforementioned ITEM which forms the corresponding theoretical framework (4, 16, 17). Within this framework, we specifically consider the “social and organizational context” and address the specificities of the less affluent county settings and China’s ongoing national health reforms.

Team composition and individual characteristics are important team inputs that are well researched in China but not for county-level hospitals (8). These inputs therefore need to be explicitly addressed. The Chinese culture emphasizes the hierarchy in organizations (18, 19), which implies that “leadership” is an important teamwork input and process worthy of special attention. Finally, we are especially interested in exploring team processes, as they have thus far received little attention in Chinese health services research on teamwork (8).

The topic list for team interventions studied in part two contains the aforementioned categories “training”, “tools”, and “(re)design” (5). Furthermore, as the Chinese government promotes the development of multidisciplinary teams (MDTs) and requires county-level, provincial level, and national level hospitals to establish MDTs, MDTs receive special attention within the category “(re)design” (20).

### Inclusion criteria and sampling

We consider a hospital to be a county-level hospital if it is located in a county or in a county-level city in China. We consider a county or county-level city to be less affluent if its GDP *per capita* level was below the national average in 2020, i.e., 72,447 Chinese Yuan (10,154 US Dollars) (21). We initially selected 15 county-level hospitals from areas thus identified as less affluent by purposive convenience sampling with the aid of the Health Human Resources Development Center of the National Health Commission of China and the Health County Media (22). The research team has no direct connections with these studied hospitals. In addition, the first author is from China and has worked as a health care professional in China for several years, so he well knows the Chinese health system and the context of this study, which will be helpful for conducting the study and analyzing data.

From each county-level hospital, we intended to interview the hospital president and one team leader who was in turn proposed by hospital senior management. The reason for enrolling hospital presidents and team leaders is that they, as both health care professionals and managerial personnels, most clearly know the influence of the unique context (i.e., county-level hospitals in less affluent areas of China) on team functioning and will provide the most valuable perspectives for this study. Data saturation determined the final sample size as we checked for saturation (i.e., all relevant themes were identified, and the same themes repeatedly emerged.) after conducting interviews with the respondents from hospitals in the initial set (23, 24).

### Data collection

Ultimately, 30 interviews were conducted *via* WeChat voice calls between September and December 2021. These interviews lasted from 38 min to 79 min and were recorded for further analysis. The first author transcribed all the audio-recordings in Chinese, translated 5 Chinese transcripts into English to be used for the independent coding process and alignment of the codes between the first and second author, and pseudonymized them to protect participants’ privacy.

### Data analysis

A thematic analysis was conducted via the software Atlas.ti and Microsoft Excel to generate codes and themes (25). The data analysis is characterized by a combination of an inductive and deductive approach (26, 27). The first and second authors independently analyzed and coded the English transcripts. While the interview questions were partly based on theory, we primarily used open coding in the data analysis (following an inductive approach). During the coding process, the first and second authors first familiarized themselves with the transcripts and created preliminary codes. Thus, these codes primarily emerged inductively from our data. Further synthesis of the codes also adopted a deductive approach when interpreting and reflecting from the perspectives of the theories used to generate the interview guide (4, 5, 16, 17).

After the preliminary coding process was finished, the first and second authors compared and discussed dissimilarities in their independent codes until consensus was reached. Then, the first author continued analyzing and coding the remaining Chinese transcripts based on the preliminary codes. After the coding for all transcripts was completed, discussion took place again between the first and second authors to resolve any issues with the codes. Next, themes were derived from these revised codes and subsequently merged into several overarching themes. These overarching themes were discussed and revised multiple times among all the authors in the process of data synthesis and developing the results section until consensus was reached. This triangulation of researchers ensures the rigor, credibility, and reliability of the study.

## Results

**Figure 1:**
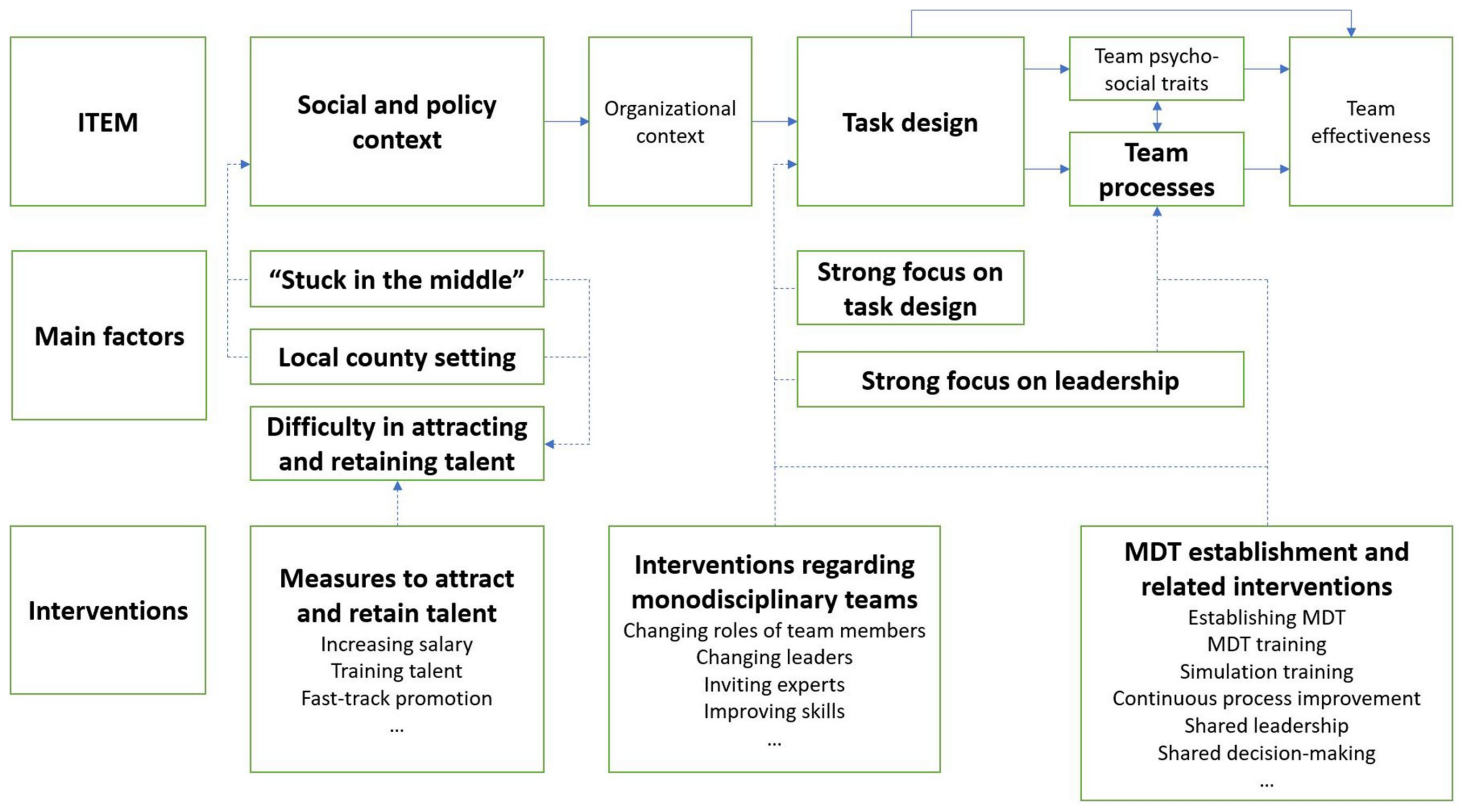
Overview of the results.

The data obtained during part one of the interviews, which addresses the first research question, yielded five main factors that influence team functioning in county-level hospitals in less affluent areas of China. These main factors are “stuck in the middle”, local county setting, difficulty in attracting and retaining talent, strong focus on task design, and strong focus on leadership. The results for part two which addresses the second research question on team interventions are presented subsequently. The overview of the results is shown in Figure 1. Dash lines and arrows indicate the connection between the ITEM and the findings of this study.

## The main factors that influence team functioning

### “Stuck in the middle”

From the interviews, we learn that county-level hospitals are viewed as “stuck in the middle” between primary care institutions on the one hand, and national and provincial level hospitals on the other hand. Primary care is seen as the main point of access for patients with mild diseases, whereas patients with more severe and complex conditions prefer to visit national or provincial level hospitals. County-level hospitals are, however, expected to contribute to servicing both types of patients, which puts them in a difficult position.

*“There is a very important responsibility for county-level hospitals. We have to treat not only common and frequently occurring diseases but also emergency cases and critically ill patients.”*

Moreover, the reputation of county-level hospitals is perceived as poor, which further exacerbates the difficulties in attracting patients and continuing providing health services for severe and complex patients.

*“Many patients who are critically ill, such as with cancer, have been more willing to visit national or provincial level hospitals instead of staying here.”*

As a consequence, health care professionals in county-level hospitals have few opportunities to practice all of their discipline-specific clinical skills, which makes it hard to maintain or improve the abilities of health care teams to provide appropriate care for complex cases.

*“Patients, such as those with tumors, will go to the hospitals in the prefecture-level city or even Beijing and Tianjin when they are diagnosed with tumors. You cannot retain such patients, so it is difficult to improve the clinical skills of the team.”*

Another consequence brought by the poor reputation of county-level hospitals is the lack of revenue. As county-level hospitals mainly earn their income by providing patient care, their poor reputation may negatively impact patient volumes and subsequently available financial resources. This inhibits these hospitals from buying necessary equipment for health services and can negatively impact the salary budget. When this translates into lower salaries, fewer professionals, or both, it can in turn negatively impact team functioning, health services provisioning, hospital reputation, and income, causing these hospitals to feel even more stuck.

*“The insufficiency of funding is very normal. …… First, …… you cannot carry out some health services without necessary equipment, so you are not able to treat patients. Another thing is the motivation and incentives. …… If you cannot provide enough salary, staff are not able to work well as they need to live and support their families.”*

### Local county setting

Our respondents tell us that county-level hospitals are located in specific local county settings. These areas are typically more mono-cultural than China’s big cities and have their local customs, norms, values, and dialects. Furthermore (close) interpersonal relationships are likely to exist outside of work among staff of county-level hospitals because counties and county-level cities are relatively small. The shared cultural background and social relations facilitate communication and teamwork, according to the respondents.

*“A good thing is that this is a small place, so everyone is familiar with each other. There are many social relationships behind us. … Therefore, various communication modes exist in a team.”*

However, these local county characteristics can cause integration difficulties for nonlocals, as they may have different working habits or struggle to understand the local dialect. Rather than creating an open environment for “outsiders,” county-level hospitals are often prone to recruiting local professionals.

*“We mainly recruit local employees whose families and social relationships are in our county. These employees can adapt well to our local culture and customs. Outsiders really do not fit in.”*

### Difficulty in attracting and retaining talent

As a result of the two aforementioned factors, most county-level hospitals have experienced difficulties in attracting and retaining talent. Talented professionals are reported to be likely to leave as they find it difficult to improve their clinical skills due to the lack of complex cases. The aforementioned limitations in salary budgets may further add to these challenges and cause talented professionals to seek alternative employment elsewhere.

*“There are not many patients for some disciplines, for instance, pediatrics and oncology. Then, it is hard to improve the clinical skills. The salary is also low. Therefore, they will resign.”*

Moreover, the less affluent character of the county context has further exacerbated the talent insufficiency beyond the aforementioned salary limitations.

*“Then, the living condition is also a key consideration, for example, children’s education. Nearly all the aspects here are worse than those in big cities.”*

In this case, the lack and loss of talent hinder the influx of new knowledge and skills into the health care teams in county-level hospitals, which in turn impedes effective communication between team members.

*“If the degree level of a team is too low, the acquisition and renewal of the state-of-the-art medical knowledge is limited. This will hinder the communication within a team as no one understands the latest knowledge.”*

To change this situation, county-level hospitals have taken measures in recent years to recruit young talent (The specific measures will be discussed later when reporting interventions). As a consequence, an increasing number of young health care professionals appear in county health care teams, increasing intergenerational interactions with both positive and negative consequences.

Different respondents stress the harmony and energy that young health care professionals bring to the teams, which increases the vitality within teams and is beneficial for the interaction between these team members.

*“Most of the team members are young. They are energetic. The atmosphere within the team is harmonious. Therefore, it is easy to arouse their enthusiasm for work.”*

Some older doctors are willing to teach and support their young colleagues, improving their clinical skills and the cohesion and communication within teams.

*“Young doctors are less experienced. Then, the older doctors teach them. … This is the mode of teaching and helping. Everyone feels happy to work on the team. The whole team is also harmonious.”*

However, not all older doctors are cooperative. Some feel threatened by these young professionals and refuse to share their knowledge and support their younger colleagues. As a result, these young health care professionals may experience difficulties integrating and be more likely to leave.

*“A team recruited a professional with a high degree. The older staff on the team felt threatened and did not support the professional’s work. This young talent found himself unable to use his knowledge there, so he finally left.”*

### Strong focus on task design

From the interviews, we learn that most health care teams in county-level hospitals are monodisciplinary and adopt a monodisciplinary basis for the task design within the teams.

Our respondents especially emphasize the importance of disciplinary clinical skills, team composition, and role clarity with respect to task design. Moreover, most respondents believe that clinical skills positively impact team performance.

*“It is sure that if the health care professionals’ clinical skills are better, this team will function better.”*

An appropriate team composition, for example regarding educational background and seniority, is perceived to be beneficial for team functioning by most respondents as every health care professional in the team is seen to have a well described specific role.

*“The team composition is very important. The ideal status is that old, middle-aged, and young staff should all be involved in a team. It is very helpful for team functioning.”*

### Strong focus on leadership

In addition to the importance of task design elements, most respondents also stress the pivotal role of leadership in team functioning. Team leaders must be regarded as leading experts in their field, with excellent clinical skills, for a team to function well.

*“As a team leader, he or she must be a leading expert of the discipline. Namely, his or her clinical skills are very good. If every decision and each step arranged by the team leader is reasonable, the team members will firmly support his or her leadership.”*

Furthermore, team leaders’ individual characteristics and leadership skills are seen as crucial to ensure high-quality team functioning.

*“First, a team leader should have foresight; otherwise, the team planning will be influenced. Second, he or she needs to possess executive ability. … Third, a team leader must be fair, or the team will not be cohesive. Fourth, decisiveness, which is part of decision-making, is needed for a team leader.”*

In addition, some of the respondents mention the crucial role of hospital management in team functioning. They not only monitor team functioning but are also involved in resolving operational issues and in introducing interventions.

“*The hospital administrators usually visit each health care team. … Staff can report issues to the hospital president via WeChat or telephone. Then, these issues will be solved.”*

Despite the importance of a clear hierarchy and strong leadership, most of the respondents do not think there is a substantial power distance within teams in county-level hospitals. This relates to the shared backgrounds and social ties between the team members.

*“Although the team leaders have some power and managerial ability, the power distance in our area is not that high. All the team leaders get along with team members in real daily life, so there is no barrier to the communication between team leaders and team members.”*

In particular, young leaders are seen as more open-minded and willing to listen to others.

*“The team leader and the doctors on the team are young, so there is no barrier to the communication between us. … When team members raise the issues they find, it helps the team develop or even helps the team leader better manage the team. We need to adopt their good suggestions.”*

Furthermore, a few respondents even state that managerial delegation is encouraged and supported by team leaders and seen as beneficial for team functioning.

*“A good team is a team on which everyone participates in management under the supervision of the team leader. … We have taken some measures, for example, assigning some administrators for quality control, nosocomial infection control and team operation. These people can help the team leader better manage the team. … On some specific things, team leaders do not know better than the team members.”*

### Interventions for improving team functioning

County-level hospitals have implemented different interventions to improve team functioning as addressed in the second part of the interviews. These interventions can be synthesized into three categories: measures to attract and retain talent, interventions mainly focused on monodisciplinary teams, and interventions to establish and improve MDTs.

### Measures to attract and retain talent

Facing the difficulty of attracting and retaining talent, most county-level hospitals have taken measures to reverse this situation. These measures include increasing talent salaries, sending staff to learn clinical knowledge and practice their clinical skills in national or provincial level hospitals, and promoting them to a higher professional title or managerial position at an early stage. Together, these interventions are intended to make county-level hospitals more attractive for recent university graduates.

*“If this young recruit is full of positive energy and good at every aspect of his or her job, we will promote him or her to a managerial position to stimulate his or her enthusiasm for work and let him or her see the hope to work here.”*

Interestingly, one of the hospitals in our study has introduced a form of unified personnel management to attract talent from primary care institutions. Well-performing professionals from primary care institutions have the chance to be promoted to this county-level hospital while at the same time poorly performing professionals from the county-level hospital are considered to be reemployed in primary care. This human resource management practice is perceived as effective.

*“Staff from primary care institutions can compete for the opportunity to work in our hospital. … This mode gives these staff the hope to work in better hospitals and improve their quality of life. Meanwhile, the staff in our hospital feel a sense of crisis. If they do not work well, it is also possible for them to work in primary care institutions.”*

Respondents have not been able to present evidence (beyond anecdotal evidence) on the effectiveness of any of the interventions to recruit qualified staff and mitigate their willingness to leave.

### Interventions regarding monodisciplinary teams

County-level hospitals display a preference for interventions on task design, in particular for leaders, to improve the performance of monodisciplinary teams. These interventions, for instance, include changing the roles of team members, changing leaders, and inviting experts from national or provincial level hospitals. In addition, interventions include the improvement of task related skills such as clinical skills and managerial skills.

*“If a team leader cannot help the team function well, … we will change the team leader. … We have successful examples. Some teams have obviously functioned much better after we changed their team leaders.”*

*“We usually organize training around clinical skills. For instance, cardio-pulmonary resuscitation, …, and emergency tracheal intubation. … It is very effective.”*

### Multidisciplinary team (MDT) establishment and related interventions

County-level hospitals have come to realize that the conventional monodisciplinary setting does not meet the demands of the increasing volumes of patients with complex, critical, conditions. These multimorbid conditions especially need the expertise of multiple specialties. Furthermore, the Chinese national health reforms stipulate that county-level hospitals have to establish MDTs to improve the quality of care for emergency patients and critically ill patients by introducing five MDT centers, i.e., chest pain center, stroke center, trauma center, critically ill maternal treatment center, and critically ill neonatal treatment center (20). County-level hospitals have taken up the establishment of MDTs for these centers to improve the consultation for complex cases (e.g., oncological patients) and to ensure integrated care for common conditions that require the involvement of multiple specialties (e.g., diabetes and hypertension).

As was the case for the monodisciplinary teams, task design elements regarding clinical skills, team composition, and hierarchy are stressed to be of importance for the functioning of MDTs. For example, multidisciplinary consultation teams often have a fixed composition (i.e., chief physicians and deputy chief physicians) to ensure the quality of consultation. Likewise, the leader of the core discipline of an MDT center reportedly always leads the multidisciplinary collaboration within the center. In multidisciplinary consultation teams, the most experienced doctor is typically appointed to lead and integrate the views of the team members from various disciplines.

*“Take the chest pain center as an example. The main discipline of this center is cardiology. The leader of cardiology, who is also the leader of the chest pain center, is responsible for arranging everyone’s work within the team. Other team members are in a cooperative position and should follow the team leader’s arrangement.”*

Experts from national or provincial level hospitals may be invited to help make final decisions when the team leader is not able to deal with divergent opinions within the MDT due to the limited knowledge and clinical skills in county-level hospitals.

The newly built MDTs also bring new challenges for teamwork, especially regarding collaboration. For instance, some health care professionals are reluctant to work with those from other disciplines. Therefore, in some of these cases, county-level hospitals have organized training to increase staff awareness of MDT collaboration.

*“These doctors and nurses have received specialized MDT training. Their thinking is unified, and they have awareness of MDT collaboration.”*

The multidisciplinary collaboration difficulties have caused hospital management to initiate interventions targeting the improvement of team processes (e.g., communication, collaboration, and coordination) rather than intervening in task design. Simulation training is frequently reported with the purpose of promoting the coordination and collaboration within MDTs. Most respondents perceived teamwork improvements from simulation training.

*“After the operation of the MDT and simulation, the communication and coordination between disciplines improved. … Another thing is that doctors’ and nurses’ clinical skills have also improved. … Now, they also have knowledge of other disciplines; their capabilities in their basic clinical work to treat patients have improved.”*

Furthermore, there were reports that hospital management implemented continuous improvement of MDT processes after simulation training and the initial implementation of MDTs. Shared leadership and decision-making are seen to contribute to such process improvement.

*“This is a process of gradual optimization. After the MDT collaboration, … we usually discuss the existing issues. Everyone expresses their opinions on how to optimize the procedures and workflows, how to save time and how to improve efficiency. This is what we are continually improving.”*

## Discussion

In this study, we aim to understand the main factors that influence team functioning and the interventions implemented to improve team functioning in county-level hospitals in less affluent areas of China.

These main factors are covered below following the logic of the synthesis presented in the results section. For each of the factors, we additionally discuss whether they can be viewed as facilitators, barriers, or both. The main interventions and their associated barriers are discussed next.

Respondents’ views on the factors regarding the contextual setting of the studied hospitals (i.e., the intermediate position in the Chinese health system and the local county setting) indicate that the contextual setting may bring both barriers and facilitators.

The context-specific barriers mostly relate to resource shortages such as staff shortages, lack of equipment, and insufficient funding. These resource shortages have been reported for hospitals in other low-income and middle-income countries and are seen as a barrier to health care delivery (28–30). Personnel shortages are also reported in rural areas in high-income countries (31, 32). Our results confirm that these context-related resource shortages may negatively impact health care delivery and additionally show that they may exacerbate the personnel and financial shortages. Moreover, the relatively poor living conditions provided by the less affluent settings can cause young staff to leave. All these barriers negatively influence team functioning in county-level hospitals and can cause them to be stuck even deeper between primary care and provincial and national level hospitals.

At the same time, our results reveal that the local county setting can facilitate team functioning in county-level hospitals due to the strong sense of community and shared local culture and values. This confirms previous evidence from rural areas in other countries (33, 34). These local idiosyncrasies can enhance the communication between local team members in county-level hospitals. However, we also find that local culture and values can turn into a barrier when “outsiders” may perceive it as difficult to integrate and subsequently are more likely to leave.

From the findings, we know that Chinese county-level hospitals have implemented various interventions to overcome these barriers. Fast-track promotion (i.e., promoting talent to a higher professional title or managerial position at an early stage) aims to attract and retain talent as it provides a faster career path in comparison to national and provincial level hospitals. The resulting influx of young talent may bring intergenerational differences to health care teams. The emergence of these differences was found to be a barrier and a facilitator, depending on the attitudes of older health care professionals toward their younger colleagues. We present suggestions for overcoming intergenerational barriers below when discussing interventions into team processes.

The medical treatment alliance initiated by the Chinese authorities helps county-level hospitals overcome resource shortages and improve team functioning by training staff in national or provincial level hospitals and inviting experts to support county-level hospitals (35). Our respondents provide little evidence on the effectiveness of such interventions yet, which therefore is an interesting area for future research.

The scientific literature provides suggestions for other interventions that thus far appear to have been disregarded. The integration of “outsiders” can, for instance, be promoted by diversity awareness training for team leaders and team-building exercises for team members (36, 37). Such interventions can more generally contribute to building a cohesive and inclusive organizational and team culture that facilitates attracting and retaining “outsiders” to advance hospital performance.

Our results on team interventions show that county-level hospitals prefer interventions to improve technical skills and interventions in team structure to improve team performance, especially for monodisciplinary teams. A recent systematic review on teamwork in Chinese hospitals also shows a preference for training clinical skills and redesigning team structure (8). Based on the ITEM, both technical skills and team structure belong to task design (4). It may then be noted that the identified preference to intervene in task design in Chinese county-level hospitals contrasts with the predominant focus of Western hospitals to intervene in team processes, which more frequently involve simulation training and crew resource management training and use tools for promoting and facilitating communication (5).

One explanation for this difference is that team processes such as communication and collaboration are not perceived to require improvement interventions because of the shared cultural background and close social relationships among team members. Moreover, the “collectivist” values of Chinese organizational culture may naturally facilitate cooperation within teams, thus reducing the (perceived) need to improve processes (18, 19).

Another explanation may lie in the cultural differences between China and Western countries. Chinese culture emphasizes hierarchy in organizations (18, 19), which helps clearly define the hierarchy and leadership within teams and subsequent top-down communication. As a result, teamwork problems are preferably resolved by changing the team leader or team structure rather than by intervening in team processes.

Despite the emphasis on task design interventions, team process interventions can still be valuable when issues in team processes appear to be rooted in team structure. For example, interpersonal conflicts may occur due to the intergenerational differences in values, personality, and behaviors brought by the influx of young staff, as discussed above (38–40). The literature summarizes a number of interventions for relieving such conflicts, for instance, reframing intergenerational differences, organizing team building activities, providing equal development opportunities for all generations, and facilitating communication by using other generations’ language (41, 42). To avoid and resolve potential intergenerational conflicts within teams, county-level hospitals may learn from these interventions and develop their own tailored interventions.

As the Chinese health reforms are deepening, the Chinese government has promoted “Patient-Centered Care” and advocated the establishment of MDTs in Chinese hospitals to address patients’ multimorbidity (7, 20). Successful implementation of MDTs can promote desired team and patient outcomes, such as increased team innovation capacity, reduced incidence of adverse events, and improved staff and patient satisfaction (43, 44). Compared to monodisciplinary teams, newly built MDTs were found to exhibit distinct features and confront new barriers for which different (types of) interventions are implemented.

Our findings show the difficulty of collaborating across disciplines surfaces as a main barrier to MDT effectiveness. This difficulty might be rooted in the traditional Chinese value “collectivism,” which causes professionals to commit to and behave more cooperatively with the “in-group”—their discipline—and show a corresponding tendency to disregard those outside of the “in-group”—staff from other disciplines (19, 45–47). Although MDTs are a new “group” gathering health care professionals from many disciplines, staff may continue to consider professionals from other disciplines as “out-groups” and thus be reluctant to collaborate with them in MDTs. The literature provides further evidence that language barriers between disciplines and conflicts across disciplines can form barriers to MDT collaboration (48–50).

Our results indicate that these barriers to MDT collaboration have prompted an interest in team processes, and county-level hospitals have started to implement team process interventions to improve MDT functioning. From the findings, we know that Chinese county-level hospitals have organized simulation training to promote the coordination and collaboration within MDTs. Moreover, hospital management has initiated corresponding continuous improvement of MDT processes.

The shared leadership and decision-making in such continuous improvement further strengthens our finding that the low power distance is perceived to be low in county-level hospitals, which is seen as conducive to effective teamwork by the respondents.

These interventions are broadly in line with the recent international literature on team processes and the positive impact of improving team process elements such as communication, collaboration, coordination, and decision-making on the effectiveness of MDTs (43, 51, 52).

Our findings on the main factors that influence team functioning and team interventions in county-level hospitals in less affluent areas of China may be generalized to other less affluent, non-Western contexts. However, as specific Chinese cultural traits appear to be embedded in our findings, the external validity in the aforementioned contexts may be limited.

## Limitations

There are some limitations of this study. First, all respondents have managerial roles, and we did not enroll other health care professionals. Hence, those professionals’ perspectives on team functioning are not included. Second, we selected 15 hospitals to advance the understanding of team functioning in county-level hospitals in less affluent areas of China. Larger-scale studies can provide a stronger evidence base for team functioning in county-level hospitals. Third, as we did not enroll participants from primary care institutions, national or provincial level hospitals, or hospitals in more affluent areas, it remains unclear to what extent the identified factors and interventions are specific to county-level hospitals in less affluent areas of China. Fourth, this study focused on the main factors and interventions to be particularly relevant for county-level hospitals in less affluent areas of China. Therefore, it does not provide a general analysis of teamwork and team functioning in these hospitals. Last, this study focused on factors and interventions that were commonly reported and has not analyzed differences between county-level hospitals, which may therefore be an interesting direction for future research.

## Conclusion

The specific contextual features and the focus on task design and leadership influence the functioning of health care teams in county-level hospitals in less affluent areas of China. There is a strong preference to intervene in team structure and leadership to improve team functioning. Due to the integration difficulty for “outsiders,” intergenerational interaction and the establishment of MDTs, process interventions are likely of additional benefit for county-level hospitals to improve team functioning and the quality of care. Recent initiatives in this direction are a promising area for practice and scientific research, strengthening the evidence base.

## Data availability statement

The raw data supporting the conclusions of this article will be made available by the authors, without undue reservation.

## Ethics statement

The studies involving humans were approved by The Research Ethics Review Committee of Erasmus School of Health Policy and Management, Erasmus University Rotterdam. Written informed consent was not required for this study in accordance with the local legislation and institutional requirements. The participants provided their informed consent on record to participate in this study.

## Author contributions

HW, JW, MB-S, and JK: conception or design of the work, data analysis and interpretation, critical revision of the article, and final approval of the manuscript. HW: data collection and drafting the article. All authors contributed to the article and approved the submitted version.

## Funding

This work was supported by China Scholarship Council (Grant number: 201906160092). This funder has no role in the study design, data collection, data analysis, interpretation of data, and writing the manuscript.

## Conflict of interest

The authors declare that the research was conducted in the absence of any commercial or financial relationships that could be construed as a potential conflict of interest.

## Publisher’s note

All claims expressed in this article are solely those of the authors and do not necessarily represent those of their affiliated organizations, or those of the publisher, the editors and the reviewers. Any product that may be evaluated in this article, or claim that may be made by its manufacturer, is not guaranteed or endorsed by the publisher.

## Abbreviations

ITEM: Integrated (Health Care) Team Effectiveness Model
SRQR: Standards for Reporting Qualitative Research
MDT: multidisciplinary team

## References

[1] Kohn LT Corrigan JM Donaldson MS. To err is human: Building a safer health system. Washington, DC: National Academy Press (2000). 312 p. 25077248

[2] Manser T. Teamwork and patient safety in dynamic domains of healthcare: a review of the literature. Acta Anaesthesiol Scand. (2009) 53:143–51. doi: 10.1111/j.1399-6576.2008.01717.x, PMID: 19032571

[3] Schmutz JB Meier LL Manser T. How effective is teamwork really? The relationship between teamwork and performance in healthcare teams: a systematic review and meta-analysis. BMJ Open. (2019) 9:e028280. doi: 10.1136/bmjopen-2018-028280, PMID: 31515415 PMC6747874

[4] Lemieux-Charles L McGuire WL. What do we know about health care team effectiveness? A review of the literature. Med Care Res Rev. (2006) 63:263–300. doi: 10.1177/1077558706287003 16651394

[5] Buljac-Samardzic M Doekhie KD van Wijngaarden JDH. Interventions to improve team effectiveness within health care: a systematic review of the past decade. Hum Resour Health. (2020) 18:2. doi: 10.1186/s12960-019-0411-3 31915007 PMC6950792

[6] McCulloch P Rathbone J Catchpole K. (2011). Interventions to improve teamwork and communications among healthcare staff. Br J Surg. (2011) 98:469–79. doi: 10.1002/bjs.7434 21305537

[7] World Bank, World Health Organization. Healthy China: Deepening health reform in China: Building high-quality and value-based service delivery. Washington, DC: World Bank Group (2019). Available at: https://documents1.worldbank.org/curated/en/690791553844042874/pdf/Building-High-Quality-and-Value-Based-Service-Delivery.pdf (accessed September 29, 2022).

[8] Wang H Buljac-Samardzic M Wang W van Wijngaarden J Yuan S van de Klundert J. What do we know about teamwork in Chinese hospitals? A systematic review. Front Public Health. (2021) 9:735754. doi: 10.3389/fpubh.2021.735754, PMID: 34976910 PMC8719585

[9] National Health Commission. The statistical bulletin of the development of health services in China in 2021 (2022). Available online at: http://www.gov.cn/xinwen/2022-07/12/content_5700670.htm (accessed September 29, 2022).

[10] National Bureau of Statistics of China. Statistical communique of the People’s republic of China on the 2021 National Economic and social development. (2022). Available at: http://www.stats.gov.cn/english/PressRelease/202202/t20220227_1827963.html (accessed August 25, 2023)

[11] Denyer D Tranfield D van Aken JE. Developing design propositions through research synthesis. Organ Stud. (2008) 29:393–413. doi: 10.1177/0170840607088020

[12] Rahi S. Research design and methods: a systematic review of research paradigms, sampling issues and instruments development. Int J Econ Manag Sci. (2017) 6:1–5. doi: 10.4172/2162-6359.1000403

[13] Giorgi A. The phenomenological movement and research in the human sciences. Nurs Sci Q. (2005) 18:75–82. doi: 10.1177/0894318404272112 15574702

[14] Rabionet SE. How I learned to design and conduct semi-structured interviews: an ongoing and continuous journey. Qual Rep. (2011) 16:563–6. doi: 10.46743/2160-3715/2011.1070

[15] O’Brien BC Harris IB Beckman TJ Reed DA Cook DA. Standards for reporting qualitative research: a synthesis of recommendations. Acad Med. (2014) 89:1245–51. doi: 10.1097/ACM.0000000000000388, PMID: 24979285

[16] Dietz AS Pronovost PJ Mendez-Tellez PA Wyskiel R Marsteller JA Thompson DA. A systematic review of teamwork in the intensive care unit: what do we know about teamwork, team tasks, and improvement strategies? J Crit Care. (2014) 29:908–14. doi: 10.1016/j.jcrc.2014.05.025, PMID: 25001565

[17] Korner M Butof S Muller C Zimmermann L Becker S Bengel J. Interprofessional teamwork and team interventions in chronic care: a systematic review. J Interprof Care. (2016) 30:15–28. doi: 10.3109/13561820.2015.1051616 26709985

[18] Meyer E. The culture map: Breaking through the invisible boundaries of global business. New York, NY: PublicAffairs (2014). 288 p.

[19] Hofstede Insights. Country comparison. (2022). Available at: https://www.hofstede-insights.com/contry-comparison/ (accessed September 29, 2022).

[20] National Health and Family Planning Commission, National Administration of Traditional Chinese Medicine. The action plan for further improving health services 2018–2020. (2018). Available at: http://www.nhc.gov.cn/yzygj/s3594q/201801/9df87fced4da47b0a9f8e1ce9fbc7520.shtml (accessed September 29, 2022).

[21] National Bureau of Statistics of China. Statistical communique of the People’s republic of China on the 2020 National Economic and social development. (2021). Available at: http://www.stats.gov.cn/english/PressRelease/202102/t20210228_1814177.html (accessed January 24, 2023).

[22] Etikan I Musa SA Alkassim RS. Comparison of convenience sampling and purposive sampling. Am J Theor Appl Stat. (2016) 5:1–4. doi: 10.11648/j.ajtas.20160501.11

[23] Fusch PI Ness LR. Are we there yet? Data saturation in qualitative research. Qual Rep. (2015) 20:1408–16. doi: 10.46743/2160-3715/2015.2281

[24] Guest G Bunce A Johnson L. How many interviews are enough? An experiment with data saturation and variability. Field Methods. (2006) 18:59–82. doi: 10.1177/1525822x05279903

[25] Lochmiller CR. Conducting thematic analysis with qualitative data. Qual Rep. (2021) 26:2029–44. doi: 10.46743/2160-3715/2021.5008

[26] Thomas DR. A general inductive approach for analyzing qualitative evaluation data. Am J Eval. (2006) 27:237–46. doi: 10.1177/1098214005283748

[27] Azungah T. Qualitative research: deductive and inductive approaches to data analysis. Qual Res J. (2018) 18:383–400. doi: 10.1108/QRJ-D-18-00035

[28] Grimes CE Bowman KG Dodgion CM Lavy CB. Systematic review of barriers to surgical care in low-income and middle-income countries. World J Surg. (2011) 35:941–50. doi: 10.1007/s00268-011-1010-1 21360305

[29] Lehmann U Dieleman M Martineau T. Staffing remote rural areas in middle-and low-income countries: a literature review of attraction and retention. BMC Health Serv Res. (2008) 8:19. doi: 10.1186/1472-6963-8-19 18215313 PMC2259330

[30] Saraceno B van Ommeren M Batniji R Cohen A Gureje O Mahoney J. Barriers to improvement of mental health services in low-income and middle-income countries. Lancet. (2007) 370:1164–74. doi: 10.1016/s0140-6736(07)61263-x 17804061

[31] Weinhold I Gurtner S. Understanding shortages of sufficient health care in rural areas. Health Policy. (2014) 118:201–14. doi: 10.1016/j.healthpol.2014.07.018 25176511

[32] World Health Organization. Imbalances in rural primary care: A scoping literature review with an emphasis on the WHO European region. Geneva, CH: World Health Organization (2018). Available at: https://apps.who.int/iris/handle/10665/346351 (accessed September 29, 2022).

[33] Roussi P Rapti F Kiosseoglou G. Coping and psychological sense of community: an exploratory study of urban and rural areas in Greece. Anxiety Stress Coping. (2006) 19:161–73. doi: 10.1080/10615800600593304

[34] Warburton J Winterton R. A far greater sense of community: the impact of volunteer behaviour on the wellness of rural older Australians. Health Place. (2017) 48:132–8. doi: 10.1016/j.healthplace.2017.10.005, PMID: 29121536

[35] General Office of the State Council. The guiding opinions on promoting the establishment and development of medical treatment alliances issues by the general Office of the State Council. (2017). Available at: http://www.gov.cn/gongbao/content/2017/content_5191699.htm (accessed September 29, 2022).

[36] Kim PS. Globalization of human resource management: a cross-cultural perspective for the public sector. Public Pers Manage. (1999) 28:227–43. doi: 10.1177/009102609902800205

[37] Shen J Chanda A D'netto B Monga M. Managing diversity through human resource management: an international perspective and conceptual framework. Int J Hum Resour Manag. (2009) 20:235–51. doi: 10.1080/09585190802670516

[38] Cahill TF Sedrak M. Leading a multigenerational workforce: strategies for attracting and retaining millennials. Front Health Serv Manag. (2012) 29:3–15. doi: 10.1097/01974520-201207000-00002 23050333

[39] Kupperschmidt BR. Multigeneration employees: strategies for effective management. Health Care Manag. (2000) 19:65–76. doi: 10.1097/00126450-200019010-00011, PMID: 11183655

[40] Swearingen S Liberman A. Nursing generations: an expanded look at the emergence of conflict and its resolution. The Health Care Manag. (2004) 23:54–64. doi: 10.1097/00126450-200401000-00010 15035349

[41] McGuire D By RT Hutchings K. Towards a model of human resource solutions for achieving intergenerational interaction in organisations. J Eur Ind Train. (2007) 31:592–608. doi: 10.1108/03090590710833651

[42] Urick MJ Hollensbe EC Masterson SS Lyons ST. Understanding and managing intergenerational conflict: an examination of influences and strategies. Work Aging Retire. (2017) 3:waw009–85. doi: 10.1093/workar/waw009

[43] Epstein NE. Multidisciplinary in-hospital teams improve patient outcomes: a review. Surg Neurol Int. (2014) 5:S295–303. doi: 10.4103/2152-7806.139612 25289149 PMC4173201

[44] Fay D Borrill C Amir Z Haward R West MA. Getting the most out of multidisciplinary teams: a multi-sample study of team innovation in health care. J Occup Organ Psychol. (2006) 79:553–67. doi: 10.1348/096317905x72128

[45] Gomez C Kirkman BL Shapiro DL. The impact of collectivism and in-group/out-group membership on the evaluation generosity of team members. Acad Manag J. (2000) 43:1097–106. doi: 10.5465/1556338

[46] McAtavey J Nikolovska I. Team collectivist culture: a remedy for creating team effectiveness. Hum Resour Dev Q. (2010) 21:307–16. doi: 10.1002/hrdq.20039

[47] Triandis HC. The self and social behavior in differing cultural contexts. Psychol Rev. (1989) 96:506–20. doi: 10.1037/0033-295x.96.3.506

[48] Govender D Naidoo S Taylor M. Nurses’ perception of the multidisciplinary team approach of care for adolescent mothers and their children in Ugu, KwaZulu-Natal. Afr J Prim Health Care Fam Med. (2019) 11:e1–e11. doi: 10.4102/phcfm.v11i1.1936, PMID: 31038339 PMC6489148

[49] Coombs M Ersser SJ. Medical hegemony in decision-making–a barrier to interdisciplinary working in intensive care? J Adv Nurs. (2004) 46:245–52. doi: 10.1016/j.iccn.2004.05.003 15066102

[50] Whiteside M. The challenge of interdisciplinary collaboration in addressing the social determinants. Aust Soc Work. (2004) 57:381–93. doi: 10.1111/j.0312-407x.2004.00168.x

[51] Fleissig A Jenkins V Catt S Fallowfield L. Multidisciplinary teams in cancer care: are they effective in the UK? Lancet Oncol. (2006) 7:935–43. doi: 10.1016/s1470-2045(06)70940-8 17081919

[52] Johansson G Eklund K Gosman-Hedstrom G. Multidisciplinary team, working with elderly persons living in the community: a systematic literature review. Scand J Occup Ther. (2010) 17:101–16. doi: 10.3109/11038120902978096, PMID: 19466676

