# Supplementary material for: Factors and interventions determining the functioning of health care teams in county-level hospitals in less affluent areas of China: a qualitative study
Source: Front Public Health. 2023 Sep 28;11:1082070. doi: 10.3389/fpubh.2023.1082070 (PMC10568023; doi:10.3389/fpubh.2023.1082070)
Supplement: Supplementary file 2 [file Data_Sheet_2.pdf]

Interview Guide

Start

Our study aims to understand the team functioning and team interventions in county-level hospitals in less affluent areas of China. The teams we are interested in are healthcare teams directly providing care for patients. The administrative teams, logistic teams and IT teams are not included. We want to understand the challenges teams in these hospitals face and the problems they encounter, and how hospital management facilitates these teams to improve performance.

I have a lot of questions, but we only have a limited amount of time. I hope you understand that I therefore may need to interrupt you during the interview, to ask additional questions or to steer us to another topic. I hope this is fine by you.

(1) What different types of health care teams are there in your hospital? (**General information**)

- What makes these teams different?
- Do you also have multidisciplinary teams?
- What are the disciplines included in these MDTs?

You probably have healthcare teams in your hospital that perform very well, and teams that are (were) not working that well, now or in the past.

(2) Could you give a very specific example of one team that is working well?

- Why? what makes them so good?
- Is the context in which this team works different from other teams?
- Is the composition of this team different from other teams?
- Is or was management involvement in this team different?

- Do they have specific facilities that help them?

(3) Could you give an example of a team that doesn’t (or didn’t) work that well?

- Why do you think this team is struggling?
- Is the context in which this team works different from other teams?
- Is the composition of this team different?
- Is or was management involvement in this team different?
- Do they have specific facilities that help them?
- Are they working on improvement or supported to improve?

(4) Are there specific challenges or difficulties for team functioning in county-level hospitals in less affluent areas that other hospitals face less or do not face? (**Social and organizational context**)

-How do you cope with these challenges?

(If not mentioned, ask the specific social and organizational context)

*Literature and reports tell us that the financial support of county-level hospitals is less than larger hospitals and that some even experience insufficient funds. This may also have an influence on team functioning. (**Insufficient fund**)*

(5) Do you also perceive this challenge?

- (If so) How do you cope with this challenge?
- (If not) Why do you think so?

The national health reforms include many policies that have an effect on hospitals. For example, the health insurance policy and the medical treatment alliance. These policies may

*influence physicians' behaviors and also have an impact on healthcare teams. (**Policy influence**)*

(6) Do you see any influence of such policies on teams?

- How does it affect teams in your hospital?

Every team may face challenge and situations that are more difficult than others. For example, emergency events, very complex surgeries, or very difficult, complex patients.

(7) How do you support teams in your organization to deal with these challenges? (Take the teams you mentioned in the previous questions as examples.)

(8) Have you ever organized training programs to simulate such situations? (**Training**)

- When, for whom, what are the effects?

(9) Have you ever used tools that help support or improve communication between team members? (**Tools**)

- When, for whom, what are the effects?

Examples from literature are structured communication models (description of SBAR, the information, when, by whom, how), structured debriefing and briefing template, and structured checklists to improve the communication or using IT, cards, and some other assistant tools to facilitate and trigger the communication within the team.

- Do you use such tools in your hospital?
- When, for whom, what are the effects?

(10) Have you introduced new structures or even new teams to cope with complex team challenges? (**(Re)design**)

- When, where, what are the effects?

(11) Have you also optimized the procedures or workflows to facilitate the work within the team? (**(Re)design**)

- When, for whom, what are the effects?

*Literature describes different aspects of teamwork some of which we have already discussed. For example, team composition which is the configuration of a team and the overall mix of characteristics among people. It can be everything regarding an individual team member, such as age, gender, professional titles, professional background, educational background, and personality traits. Literature tells us that the interaction between different composition attributes can influence team effectiveness. It would be good that a team consists of staff that complement each other in the above-mentioned individual features. However, it is hard to be ideal in the real life. (**Team composition**)*

(12) What are the challenges you face in your hospital with team composition?

- How do you cope with these challenges?
- (If no) Why do you think so?

*Literature shows that county-level hospitals in less affluent areas have more difficulty to attract and retain well-educated staff than national and provincial hospitals. (**Lack of well-educated staff**)*

(13) Do you also face this challenge?

- (If so) How do you cope with this challenge?
- (If not) Why do you think so?

*We know from literature that the stability of team composition has impact on team functioning. A stable team makes team members cooperate with each other more fluently. However, if a team is too stable, there will be less innovative thinking in the team, further influencing the development of the team. (**Team stability**)*

(14) Do you also perceive such challenges in your hospital?

- How do you cope with these challenges?
- Are there any teams not that stable?
- What are the challenges of these unstable teams?
- How do you cope with these challenges?

*We know from literature that Chinese hospitals are often very hierarchical, and that people lower in hierarchy often experience difficulty to speak up, therefore inhibiting teamwork. (**Speak up**)*

(15) Do you recognize this for your hospital?

- (If so) How do you cope with it?
- (If not) Why do you think so?

*We know from literature that cooperation within a team is not that easy. Two people may work well individually, but it does not mean that they also work very well when working together. The reasons are various, for example, different people have different ideas, expertise, and personality traits, one doesn't like another, or there are some objective limited factors. These reasons make cooperation within a team more difficult. (**Team processes**)*

(16) Do you recognize this for your hospital?

- In what type of teams do you experience the most challenges in team cooperation and why?

- How do you cope with these challenges?

*We especially know that multidisciplinary teams may have trouble with teamwork because of the different knowledge, skills, and viewpoints of different disciplines but also because they may not agree on who does what. (**Multidisciplinary team**)*

(17) Is this something you recognize in your hospital? Could you give concrete examples?

- How do you deal with these challenges?

*Now, Let's talk about team leaders. We find from literature that different leaders have different ways of managing a team. For example, some leaders ask team members to do as he/she says, while some other leaders listen to everyone's opinion and encourage team members to express their views. There are advantages and disadvantages of each leadership style, so it is not easy to judge which style is better. (**Leadership**)*

(18) Could you give a concrete example to describe the leadership style of the teams you mentioned previously? The well working team and the team not working that well.

- What are the roles of leaders in these two teams?
- For the well working team, did the leader perceive any challenges for managing the team?
- How did him/her deal with these challenges?
- For the team not working that well, what are the challenges for the leader in managing the team?
- How did him/her deal with these challenges?

The end

Before ending the interview, we will repeat the research aim and keep in touch with the respondent in case we need more information.

(19) Do you want to add anything that is relevant to today's topic, but we have not talked about yet? You are very welcome to contact me if you want to add something after the interview.

(20) Do you have any questions? Could I contact you if I have missed something?

Greeting

Thank you again for participating in this interview. We appreciate your contribution to our research.
